# Combined Pulmonary Fibrosis and Emphysema and Digital Clubbing

**DOI:** 10.7759/cureus.24231

**Published:** 2022-04-18

**Authors:** Taha F Rasul, Daniel R Bergholz, Randal Rovinski, Sana Gulraiz, Ernesto Fonts

**Affiliations:** 1 Department of Infectious Diseases, University of Miami Miller School of Medicine, Miami, USA; 2 Department of Allergy and Immunology, University of Miami Miller School of Medicine, Miami, USA; 3 Department of Pulmonary and Critical Care Medicine, University of Miami Miller School of Medicine, Jackson Memorial Hospital, Miami, USA; 4 School of Public Health, West Virginia University School of Medicine, Morgantown, USA; 5 Internal Medicine, Veterans Affairs Medical Center, Miami, USA

**Keywords:** multidisciplinary decision-making, multidisciplinary, delayed diagnosis, cervical spine injury, cpfe, combined pulmonary fibrosis emphysema

## Abstract

Combined pulmonary fibrosis and emphysema (CPFE) is an underrecognized syndrome that involves simultaneous restrictive-obstructive lung disease. The prognosis is poor, and it frequently occurs with comorbidities. Heavy or former smoking is a major risk factor, and computed tomography (CT) typically shows lower zone fibrosis and upper zone emphysema. Chronic respiratory failure, pulmonary hypertension, and lung carcinoma are major causes of mortality. Diagnosis of CPFE should be combined with palliative care due to the high mortality of the condition, especially in the case of delayed diagnosis. We present the case of a 73-year-old male with a history of non-small cell lung cancer, 50 pack-year smoking, and cervical spine injury (CSI) with a late diagnosis of CPFE. After presenting to the emergency department for an acute exacerbation of dyspnea and hypoxia, he was initially treated with a congestive heart failure protocol. Further examination showed mixed pulmonary function tests as well as digital clubbing, and a CT scan showed changes indicative of advanced bullous emphysema diffusely throughout both lungs with an upper lobe predominance and basilar fibrosis. He was diagnosed with CPFE and immediately treated for both restrictive and obstructive lung diseases with supplemental oxygen, albuterol, ipratropium, corticosteroids, systemic antibiotics, as well as provided with palliative consultation. His previous history and CSI delayed diagnosis, as his lung restriction was likely assumed to be from impaired chest wall mobility rather than CPFE. This case highlights the presentation of a relatively rare disease that was confounded by comorbidities.

## Introduction

Combined pulmonary fibrosis and emphysema (CPFE) is a rare condition comprised of a combination of restrictive and obstructive lung disease. The most common predisposing risk factor is exposure to cigarette smoke. Clinical symptoms most often include dyspnea due to upper lobe emphysema and lower lobe fibrosis leading to abnormalities in gas exchange [1]. Although the upper lobe emphysema and lower lobe fibrosis pattern is the most typical pattern found for CPFE, there can be variability in the distribution of pulmonary fibrosis and emphysema [2].

CPFE is generally categorized as interstitial lung disease. The American Thoracic Society and the European Respiratory Society classification of interstitial pneumonia in 2013 listed three main categories of idiopathic interstitial pneumonia: major idiopathic interstitial pneumonia, rare idiopathic interstitial pneumonia, and unclassified idiopathic interstitial pneumonia. CPFE falls into the latter "unclassified" category and was first described in 2005 [3]. 

The prevalence of this condition is not completely understood, likely due to the rarity of this condition. The pathogenesis has been unclear as well; however, there is a strong association with smoking and other environmental exposures such as mineral dust and agricultural and industrial compounds [4]. There is also a strong association with lung cancer, both as a predisposing factor and outcome [5]. Autoimmunity has also been shown to play a role in CPFE. Patients with elevated antinuclear antibodies (ANA) and the presence of autoimmune conditions demonstrated improved survival compared to those without autoimmunity [6]. 

Most authors have suggested that CPFE can be thought of as a combination of chronic obstructive pulmonary disease (COPD) and idiopathic pulmonary fibrosis (IPF). The clinical symptoms are primarily cough and dyspnea, which are nonspecific and overlap with more common symptoms of long-term smoking, such as those seen with COPD or IPF. However, there are some subtle differences [7]. COPD generally has a productive cough which is chronic but has variable sputum production. There is also an element of progressive dyspnea. The cough and sputum production generally occur before the airflow limitation, usually preceding it by a few years. As for IPF, the onset and progression of the disease are more rapid, and dyspnea is typically the initial symptom and reported in up to 90% of patients with IPF [8]. The dyspnea is also frequently accompanied by a dry and nonproductive cough. CPFE involves elements of both COPD and IPF; however, the presenting symptoms are closer to those of pulmonary fibrosis as compared to those of COPD. Progressive shortness of breath generally predominates as the most common initial symptom. CPFE also has a disproportionately high prevalence of concomitant pulmonary hypertension compared to COPD or IPF individually [9]. 

Diminished diaphragmatic motion during deep breathing can also be an initial presentation of CPFE. With such a diverse array of presentations, the CPFE syndrome has a poor prognosis with a five-year survival of approximately 50-60%, depending on comorbidities and extent of pulmonary disease. There are currently no specific treatments or established guidelines for CPFE [10]. Generally, the mainstay would include the management of pulmonary fibrosis and emphysema separately. The following case represents a patient with an extensive medical history who suffered from sudden-onset dyspnea of unclear etiology.

## Case presentation

A 73-year-old male with an extensive medical history of a cervical spine injury (leading to quadriplegia), non-small cell lung cancer treated with lobectomy eight years prior, COPD, hypertension, type 2 diabetes mellitus, 50 pack-year smoking, post-traumatic stress disorder (PTSD), and peripheral neuropathy was brought to the emergency department for acute-onset hypoxia. His caretaker reported that his oxygen saturation was approximately 75%, and he had been experiencing progressive dyspnea. He usually relied on a two-liter nasal cannula but was experiencing respiratory distress even with the supplemental oxygen. He was afebrile and had no other major complaints, such as chest pain, diarrhea, nausea, and vomiting. Upon arrival at the emergency department, he was provided with a six-liter nasal cannula which initially resolved his dyspnea. His dyspnea progressively worsened over the next four hours, and he was initiated on a high flow nasal cannula at 35 L/minute, and a fraction of inspired oxygen (FiO_2_) of 50-60% was required to keep his oxygen saturation above 88%. Physical examination was notable for bilateral digital clubbing, bibasilar crackles, and bilateral leg swelling. Other relevant laboratory findings such as troponins, electrocardiogram (ECG), and a metabolic panel were within normal limits. However, a chest X-ray displayed changes indicative of cardiomegaly without any patterns suspicious of pneumonia. After stabilization, treatment was initiated for a presumed congestive heart failure exacerbation, including diuresis with furosemide. An echocardiogram was notable for right ventricular hypertrophy (5.3 mm wall thickness) and septal dyskinesia.

There was a distinct lack of improvement in symptoms, even after his apparent volume overload was treated over the course of the next day. Further differential diagnoses included pulmonary embolism, COPD exacerbation, and pneumonia, and he was treated empirically with bronchodilators, antibiotics, and corticosteroids as well. Pulmonary function tests (PFTs) demonstrated reduced forced expiratory volume in one second (FEV1; 79% of expected), reduced diffusion of carbon monoxide (DLCO) at 14 mL/mmHg/min (reference values between 18.6 and 34.6), and an overall mixed pattern of restriction and obstruction. FEV1 to forced vital capacity (FVC) ratio was 87% of expected (Table 1). 

**Table 1:** Spirometry taken three months before hospital admission Relatively preserved FEV1/FVC is coupled with significantly reduced FEF25-75%, suggesting a mixed restrictive-obstructive pattern. FVC - forced vital capacity; FEV1 - forced expiratory volume in one second; FEF25-75% - mid-expiratory flow rate; PEF - peak expiratory flow rate

Spirometry	Reference average (reference ranges)	Pre-bronchodilators (% of reference average)	Post-bronchodilators (% of reference average)
FVC (liters)	4.4 (3.3 - 5.6)	3.9 (88)	4.1 (92)
FEV1 (liters)	3.4 (2.6 - 4.3)	2.6 (77)	2.7 (79)
FEV1/FVC (%)	78 (69.4 - 86.0)	67 (87)	67 (87)
FEF25-75% (liters/sec)	3.2 (1.5 - 4.9)	1.4 (43)	1.3 (40)
PEF (liters/sec)	8.2 (4.3 - 12.0)	8.0 (98)	7.9 (97)

**Figure 1:**
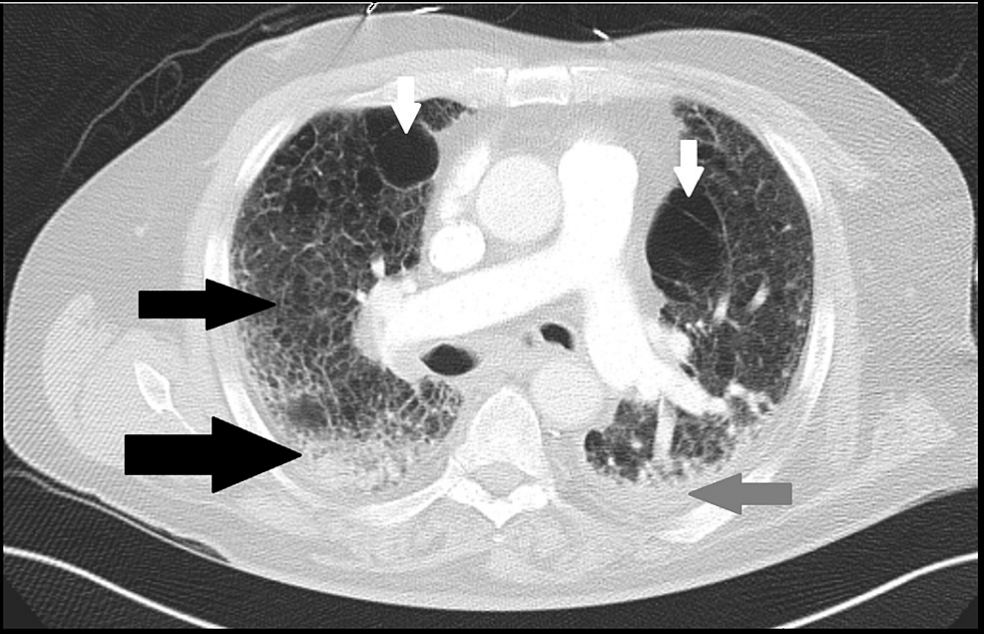
Computed tomography of basilar lungs CT of basilar lungs shows left pleural effusion (grey arrow), pulmonary fibrotic 'honeycombing' pattern (black arrows), residual bullous changes (white arrows), and peripulmonary artery lymphadenopathy.

A computed tomography (CT) of the chest was performed and showed moderate to advanced pulmonary interstitial edema, fibrosis, and a small left pleural effusion (Figure 1).

**Figure 2:**
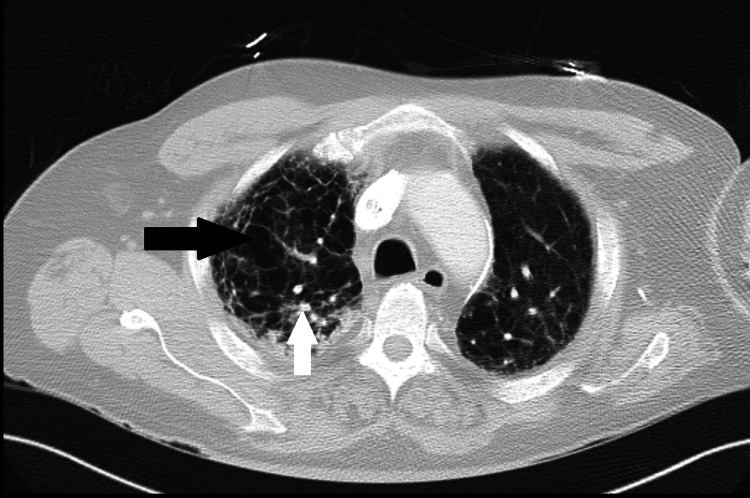
Computed tomography of the upper-middle lung Upper-middle lung CT shows diffuse emphysematous changes (black arrow) with a bullous disease, as well as calcified nodules (white arrow).

Additionally, changes indicative of advanced bullous emphysema were present diffusely throughout both lungs with an upper lobe predominance (Figure 2).

A subsequent CT chest with pulmonary angiography confirmed the lack of pulmonary emboli as well as prominent cardiomegaly, severe pulmonary fibrosis, and mediastino-hilar lymphadenopathy. After examination of the history, PFTs, and CT findings, it was determined that our patient's clinical presentation best fit the diagnosis of CPFE syndrome. Palliative care was consulted, owing to the high risk of mortality in this patient. His CPFE was further managed by approaching the obstructive and restrictive aspects separately. Bronchodilators (including albuterol and ipratropium), antibiotics (amoxicillin-clavulanate), supplemental oxygen, and corticosteroids were used to manage the obstructive symptoms, whereas judicious fluid management helped manage the restriction. His condition improved over the next few days, whereby he was able to be weaned down to one-liter supplemental oxygen. Palliative experts were also consulted and separately evaluated the patient. He opted to continue medical management of his CPFE, understanding the prognosis of his disease.

## Discussion

Based on the clinical and imaging criteria, our patient with combined restrictive and obstructive lung disease had CPFE. Since the most accurate confirmatory test is a chest CT, the combined pattern of upper lobe fibrosis and lower lobe bullous emphysema was key in establishing the diagnosis. This patient, who was paraplegic due to his cervical spine injury, was also expected to have a restrictive lung disease secondary to decreased chest wall mobility. The delay in diagnosis could have been due to the fact that an extensive smoking history combined with a recalcitrant chest wall could also create a combined restrictive-obstructive clinical picture. Additionally, the lack of confirmatory CT findings would also lead to a delay in finding the root cause. Even with a diagnosis such as CPFE, treatment is in the form of managing symptoms individually [11]. For example, his dyspnea had to be addressed with a COPD treatment (antibiotics and corticosteroids), and hypoxia was managed with high-flow oxygen. The restrictive nature of his lung disease meant that complications like cor pulmonale could occur as a result and present with symptoms of heart failure. Additionally, the echocardiogram demonstrated septal dyskinesia and right ventricular thickening, suggesting right-heart strain, which made lung-induced right heart failure likely. This is ostensibly why his initial exacerbation demonstrated peripheral edema, bibasilar crackles, and cardiomegaly. 

Most patients suffering from CPFE have a mixed pattern in pulmonary function testing. They also display marked reductions in diffusing capacity of carbon monoxide (DLCO) [12]. Perhaps the most specific diagnostic measure of CPFE is high-resolution CT. The concomitant presence of upper lobe emphysema test changes and lower lobe fibrotic changes are a reasonable starting point for suspicions of CPFE. The upper lobe emphysematous changes can include centrilobular and paraseptal emphysema. There can also be the presence of thick-walled cystic or bullous lesions, such as those seen in our patient (Figure 2). The lower lobe fibrotic changes generally include honeycombing and bronchiectasis (Figure 1). Antifibrotic drugs such as pirfenidone which have been used to treat idiopathic pulmonary fibrosis, are still undergoing trials for CPFE treatment, currently with unclear benefits [4,13].

Findings on CT for CPFE are generally heterogeneous and exist on the spectrum both in terms of the characteristics of the findings as well as the severity [13]. Pulmonary function tests can also guide diagnosis due to the combined restrictive and obstructive nature of the disease [14]. Many studies have shown that the forced vital capacity and total lung capacity are usually within normal ranges, but the DLCO is diminished significantly. This can be thought of as the counterbalance in terms of the hyperinflation found with emphysema compared to the restriction of pulmonary fibrosis, with the net result being relatively the same lung volumes as a baseline. The concurrent presence of upper airway obstruction has also been found to be a negative prognostic indicator for lung function [15].

This rare case demonstrates a rare condition that can easily be confused with other disease processes, especially in the case of an extensive medical history such as our patient. The exact prevalence of CPFE is not known. Some estimates state that in asymptomatic smokers, roughly 3% of patients may have findings indicative of CPFE [16]. Although there has been a well-established relationship between CPFE and smoking, it remains to be seen how often (and with what level of severity) it occurs in the general population. Additionally, the previous history of lung cancer, as well as resection, could likely have worsened the overall pulmonary function status of the patient, even though the pulmonary function tests showed a stable restrictive-obstructive picture [17]. According to a study conducted in 2016, resection of lung cancer in patients with preexisting CPFE led to worse outcomes and poorer prognosis compared to no resection. This means that if our patient's CPFE occurred before his left lower lobectomy, we would expect a poorer overall prognosis and potentially transition care to palliative or hospice. Another consideration is the presence of digital clubbing on physical examination, which has been associated with a higher mortality rate in CPFE patients [18].

The decision to pursue purely comfort measures is often seen in CPFE patients due to their decreased life expectancy and often the presence of comorbidities. By some estimates, the five-year survival rate of patients diagnosed with CPFE is between 35-80%, with aggressive treatment marginally improving mortality while also significantly decreasing quality of life and comfort [18]. Patients who may have an underlying cause of decreased chest wall mobility, such as obesity (or, in the case of this patient, paraplegia), should receive a further evaluation by means of a CT scan to better ascertain the cause of their dyspnea. Before escalating care to imaging, pulmonary function testing (PFTs) should demonstrate a mixed pattern of lung disease. The combination of CT findings (upper lobe emphysema, lower lobe fibrosis) combined with mixed PFTs should lead clinicians toward diagnosing CPFE.

## Conclusions

Combined pulmonary fibrosis and emphysema (CPFE) is an under-documented disease with a characteristic restrictive-obstructive presentation. It is a combination of emphysematous changes in the upper lungs and fibrotic changes in the basilar lung fields. It has a strong association with smoking, and complications can include pulmonary hypertension, lung cancer, and respiratory insufficiency. Due to the severity of the disease, the prognosis is poor, and newly diagnosed patients should be counseled on palliative or hospice care. Comorbidities such as cervical spine injuries can act as confounders and delay the diagnosis of CPFE due to the inherent disruption of chest wall function. Keeping CPFE on the differential may be appropriate in patients with respiratory exacerbations and a history of smoking. Pulmonary function tests and computed tomography are the most accurate confirmatory studies for this condition. Physical examination findings like digital clubbing are associated with a worse prognosis.
